# Previable Preeclampsia Diagnosed by Renal Biopsy in Setting of Novel Diagnosis of C4 Glomerulopathy

**DOI:** 10.1155/2017/8698670

**Published:** 2017-07-04

**Authors:** Jessica Parrott, Timothy A. Fields, Marc Parrish

**Affiliations:** ^1^Division of Maternal Fetal Medicine, Department of Obstetrics and Gynecology, University of Kansas School of Medicine, 3901 Rainbow Boulevard, Kansas City, KS 66160, USA; ^2^Department of Pathology and Laboratory Medicine, University of Kansas School of Medicine, 3901 Rainbow Boulevard, Kansas City, KS 66160, USA

## Abstract

**Background:**

Preeclampsia diagnosed before 20 weeks' gestational age is a rare entity, particularly without any predisposing factors. We report a case of preeclampsia occurring prior to 20 weeks' gestational age in the setting of a novel diagnosis of C4 glomerulopathy.

**Case:**

A G3P0020 at 18 weeks presented with new onset hypertension and proteinuria, requiring multiple antihypertensive agents to maintain control. Renal biopsy demonstrated thrombotic microangiopathic lesions and glomerular endotheliosis. C4-dominant staining and numerous subendothelial and mesangial electron dense deposits were found within the glomerulus. With no other definable etiologies, preeclampsia was diagnosed. She developed posterior reversible encephalopathic syndrome and pregnancy termination was recommended.

**Conclusion:**

The lectin complement pathway may play a role in the pathophysiology of severe, early onset preeclampsia. Renal biopsy may play an integral role in diagnosis.

## 1. Introduction

Preeclampsia is a diagnosis marked by new onset hypertension and proteinuria with usual onset after 20 weeks' gestational age. Previable preeclampsia is a rare entity with most reported cases associated with trophoblastic disease, triploidy, and antiphospholipid antibody syndrome [1–5]. Only a few published case reports exist describing onset of “pure” preeclampsia, with no identifiable predisposing factors other than advanced maternal age, at less than 20 weeks [6, 7]. We report a case of previable preeclampsia occurring prior to 20 weeks' gestation in a patient with a novel diagnosis of C4 glomerulopathy.

## 2. Case Report

A 27-year-old, gravida 3 para 0020, was transferred to our institution at 18 weeks' gestational age with new onset severe hypertension (blood pressure > 180/100 mmHg) and >10 g of protein on spot urine. Upon arrival to the labor and delivery unit, she was started on intravenous magnesium sulfate for eclampsia prophylaxis. Intermittent intravenous antihypertensive treatment was required for severe hypertension (blood pressure > 160/105 mmHg) but she was overall controlled with oral labetalol and nifedipine. Her 24-hour urine protein was 8822 mg. Her serum labs were unremarkable with a hemoglobin of 10.6 gm/dL, platelet count of 183 K/UL, creatinine of 0.6 mg/dL, AST/ALT of 24/15 U/L, and uric acid of 4.4 mg/dL. Nephrology was consulted and an extensive work-up was done. A low complement C4 of <8.0 was found. The patient, otherwise, had a negative renal ultrasound, antiphospholipid antibody studies, antinuclear antibody, C-ANCA/P-ANCA, myeloperoxidase antibody, serine protease 3 antibody, glomerular basement membrane antibody, viral hepatitis panel, thyroid stimulating hormone (TSH), and urine drug screening. Due to concern for preexisting renal pathology, the decision was made to proceed with a renal biopsy.

**Figure 1:**
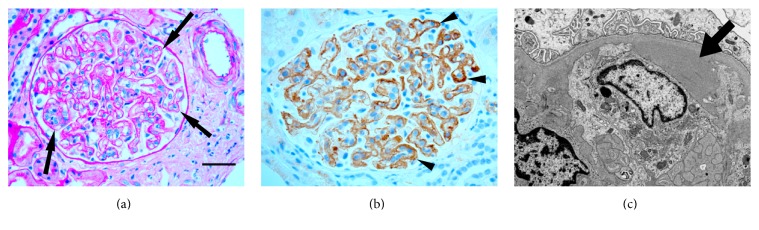
Some key renal biopsy findings. (a) PAS-stained section showed (arrows) duplication of glomerular basement membranes and endotheliosis (PAS stain; bar = 50 *μ*m). (b) Immunohistochemical stain demonstrated C4d deposition within glomerular capillaries (highlighted by arrowheads). (c) Electron microscopy demonstrated conspicuous subendothelial electron dense deposit (arrow).

Initial light microscopy findings were consistent with a thrombotic microangiopathic lesion, compatible with preeclampsia. On review with the pathologist, the main finding was glomerular endotheliosis and glomerular capillary double contours (Figure 1). There was no artery or arteriole involvement and there was no thrombi or red blood cell fragmentation. Immunofluorescence microscopy showed weak (1+) segmental glomerular staining for IgM and C1q and no staining for IgG, IgA, C3, or *κ* or *λ* light chains. The biopsy findings, the new onset severe hypertension, and new onset nephrotic range proteinuria in the absence of any other definable etiology led us to the eventual diagnosis of preeclampsia.

After a few days, the patient began to develop worsening hypertension despite being on increasing doses of oral labetalol and nifedipine, requiring additional intravenous antihypertensives for breakthrough severe range blood pressures. She also developed persistent neurologic symptoms consistent with posterior reversible encephalopathic syndrome. Her serum labs, including creatinine, remained stable. Due to the severe nature of her preeclampsia at a previable gestational age, pregnancy termination was recommended. Within 6 hours of the patient's delivery, she no longer required intravenous antihypertensive therapy. Her neurologic symptoms resolved within 72 hours of delivery. She had a follow-up visit with nephrology 17 days after delivery and her urinalysis demonstrated no protein. Her serum labs at that time included hemoglobin of 12.0 gm/dL, platelet count of 307 K/UL, creatinine of 0.74 mg/dL, and AST/ALT of 25/17 U/L.

Following her delivery, electron microscopic examination of the biopsy revealed numerous subendothelial and mesangial electron dense deposits within the glomerulus. Subsequent immunostain for C4d demonstrated intense staining of the glomerular capillaries. These findings were felt to be consistent with C4 glomerulopathy.

## 3. Comment

Preeclampsia is generally thought of as a disease that develops after 20 weeks' gestation; however, case reports have been published describing onset prior to the 20 weeks. There have been 4 cases described in the setting of pregnancy complicated by trophoblastic disease or triploidy, as well as one case complicated by antiphospholipid antibody syndrome [1–5]. Two additional cases, in women of advanced maternal age, have been described—being coined “pure” preeclampsia due to the lack of any identifiable etiology or predisposing factor. In both cases, the women had a history of preeclampsia in a prior pregnancy and one of the women had known chronic hypertension [6, 7]. These unique cases present many challenges as we must consider all diagnostic possibilities—such as lupus nephritis, hemolytic-uremic syndrome, antiphospholipid antibody syndrome, or thrombotic thrombocytopenic purpura [8]. In these difficult scenarios, renal biopsy can be a safe and useful tool in identifying the correct diagnosis and guiding appropriate management.

On renal biopsy, the most common pathologic findings in preeclampsia include endotheliosis, podocyte vacuolation, mesangial cell proliferation, and renal tubule protein casts; in addition, immunofluorescence microscopy is commonly positive for multiple complement components (C3, C1q, and C4d) and immunoglobulins (usually IgM) [9]. The findings of C4-dominant staining and numerous electron dense deposits in this case were unusual. A recently published article described 3 patients with similar findings and proposed a novel diagnosis for this entity, C4 glomerulopathy [10]. The presence of glomerular C4d, a split product of C4, is indicative of C4 activation, which can occur as a result of activation of either the classical complement pathway or the lectin pathway. Immune complex glomerulonephritides (e.g., lupus) often activate the classical pathway activation by the binding of C1q to the antibody-containing immune complexes, which can result in glomerular deposition of immunoglobulins, C3, C1q, and C4d. In the lectin pathway, lectin proteins bind to sugars (mannose) located on the microbial membranes or carbohydrate structures, which in turn activate C4 and bypass C1q activation [10]. In C4 glomerulopathy, the presence of bright C4d and negative or minimal staining for immunoglobulins, C1q, and C3 is suggestive of a lectin pathway abnormality [11]. The question is as follows: How does the lectin pathway get activated?

The currently proposed theories have included genetic factors, acquired autoantibodies, or even a paraprotein that interferes with the lectin pathway that may play a role in the development of C4 glomerulopathy [9, 12]. Lectin pathway activation has been demonstrated in some immune complex glomerular diseases, including poststreptococcal glomerulonephritis and IgA nephropathy, though those diseases have a very different microscopic appearance. In line with these proposed mechanisms, an extensive work-up was done in this patient to determine whether there were any underlying processes present that would have led to abnormal activation of the lectin pathway or other channels in the complement pathway. As part of this work-up she had negative screening for immune and infectious etiologies, in addition to a normal serum protein electrophoresis, a negative paraprotein analysis, and normal light chain analysis. We also assessed different components of her complement system including C3 and Factor H and Factor B levels, which were all normal. Her serum C4 level, which was low during the time of her initial disease presentation, normalized three weeks after her delivery. If it is not a preexisting entity, then one may speculate that there is a component of pregnancy and/or a mechanism of preeclampsia that activates the lectin complement pathway.

To our knowledge, there are only four other known cases of a pregnancy being complicated by C4 glomerulopathy, and unfortunately the current status of the patients and the outcomes of any subsequent pregnancies are unknown [9, 10, 13]. With little known information on the physiology and mechanism of lectin complement pathway activation, it is difficult to counsel the patient on risk of recurrence of such an adverse pregnancy outcome in a subsequent pregnancy. Using the best available data, we quoted a recurrence risk of 50–60% but also emphasized that there was approximately a 30% chance that the disease onset could be prior to 28 weeks' gestational age [14–16].

## References

[1] Rahimpanah F., Smoleniec J. (2000). Partial mole, triploidy and proteinuric hypertension: Two case reports. *Australian and New Zealand Journal of Obstetrics and Gynaecology*.

[2] Brittain P. C., Bayliss P. (1995). Partial hydatidiform molar pregnancy presenting with severe preeclampsia prior to twenty weeks gestation: a case report and review of the literature. *Military Medicine*.

[3] Alsulyman O. M., Castro M. A., Zuckerman E., McGehee W., Goodwin T. M. (1996). Preeclampsia and liver infarction in early pregnancy associated with the antiphospholipid syndrome. *Obstetrics and Gynecology*.

[4] Craig K., Pinette M. G., Blackstone J., Chard R., Cartin A. (2000). Highly abnormal maternal inhibin and *β*-human chorionic gonadotropin levels along with severe HELLP (hemolysis, elevated liver enzymes, and low platelet count) syndrome at 17 weeks' gestation with triploidy. *American Journal of Obstetrics and Gynecology*.

[5] Nugent C. E., Punch M. R., Barr M. (1996). Persistence of partial molar placenta and severe preeclampsia after selective termination in a twin pregnancy. *Obstetrics & Gynecology*.

[6] Hazra S., Waugh J., Bosio P. (2003). 'Pure' pre-eclampsia before 20 weeks of gestation: a unique entity. *BJOG: An International Journal of Obstetrics and Gynaecology*.

[7] Thomas W., Griffiths M., Nelson-Piercy C., Sinnamon K. (2012). Pre-eclampsia before 20-week gestation: diagnosis, investigation and management. *Clinical Kidney Journal*.

[8] Sibai B. M., Stella C. L. (2009). Diagnosis and management of atypical preeclampsia-eclampsia. *American Journal of Obstetrics and Gynecology*.

[9] Han L., Yang Z., Li K. (2014). Antepartum or immediate postpartum renal biopsies in preeclampsia/eclampsia of pregnancy: new morphologic and clinical findings. *International Journal of Clinical and Experimental Pathology*.

[10] Sethi S., Quint P. S., O'Seaghdha C. M. (2016). C4 glomerulopathy: A disease entity associated with C4d deposition. *American Journal of Kidney Diseases*.

[11] Sethi S., Sullivan A., Smith R. J. H. (2014). C4 dense-deposit disease. *New England Journal of Medicine*.

[12] Espinosa M., Ortega R., Sanchez M. (2014). Association of C4d Deposition with Clinical Outcomes in IgA Nephropathy. *Clinical Journal of the American Society of Nephrology*.

[13] Ali A., Schlanger L., Nasr S. H., Sethi S., Gorbatkin S. M. (2016). Proliferative C4 dense deposit disease, acute thrombotic microangiopathy, a monoclonal gammopathy, and acute kidney failure. *American Journal of Kidney Diseases*.

[14] Sibai B. M., Mercer B., Sarinoglu C. (1991). Severe preeclampsia in the second trimester: recurrence risk and long-term prognosis. *American Journal of Obstetrics and Gynecology*.

[15] van Rijn B. B., Hoeks L. B., Bots M. L., Franx A., Bruinse H. W. (2006). Outcomes of subsequent pregnancy after first pregnancy with early-onset preeclampsia. *American Journal of Obstetrics and Gynecology*.

[16] Bramham K., Briley A. L., Seed P., Poston L., Shennan A. H., Chappell L. C. (2011). Adverse maternal and perinatal outcomes in women with previous preeclampsia: a prospective study. *American Journal of Obstetrics and Gynecology*.

